# MMP-13 is constitutively produced in human chondrocytes and co-endocytosed with ADAMTS-5 and TIMP-3 by the endocytic receptor LRP1

**DOI:** 10.1016/j.matbio.2016.03.007

**Published:** 2016-12

**Authors:** Kazuhiro Yamamoto, Hiroshi Okano, Wakako Miyagawa, Robert Visse, Yasuyuki Shitomi, Salvatore Santamaria, Jayesh Dudhia, Linda Troeberg, Dudley K. Strickland, Satoshi Hirohata, Hideaki Nagase

**Affiliations:** a Kennedy Institute of Rheumatology, Nuffield Department of Orthopaedics, Rheumatology, and Musculoskeletal Sciences, University of Oxford, Oxford, UK; b Department of Molecular Biology and Biochemistry, Okayama University Graduate School of Medicine, Dentistry and Pharmaceutical Sciences, Okayama, Japan; c Department of Clinical Sciences and Services, Royal Veterinary College, Herts, UK; d Center for Vascular and Inflammatory Diseases, University of Maryland School of Medicine, Baltimore, USA

**Keywords:** ADAMTS, adamalysin-like metalloproteinase with thrombospondin motifs, DMEM, Dulbecco's modified Eagle's medium, EEA1, early endosome antigen 1, ECM, extracellular matrix, FBS, foetal bovine serum, Hpx, hemopexin domain, LDL, low-density lipoprotein, LRP, LDL receptor-related protein, MMP, matrix metalloproteinase, OA, osteoarthritis, RAP, receptor-associated protein, RPLP0, the 60S acidic ribosomal protein P0, TIMP, tissue inhibitor of metalloproteinases, Cartilage, Endocytosis, Extracellular trafficking, Matrix metalloproteinases, Collagenase, Osteoarthritis

## Abstract

Matrix metalloproteinase 13 (MMP-13) degrades collagenous extracellular matrix and its aberrant activity associates with diseases such as arthritis, cancer, atherosclerosis and fibrosis. The wide range of MMP-13 proteolytic capacity suggests that it is a powerful, potentially destructive proteinase and thus it has been believed that MMP-13 is not produced in most adult human tissues in the steady state. Present study has revealed that human chondrocytes isolated from healthy adults constitutively express and secrete MMP-13, but that it is rapidly endocytosed and degraded by chondrocytes. Both pro- and activated MMP-13 bind to clusters II and III of low-density lipoprotein (LDL) receptor-related protein 1 (LRP1). Domain deletion studies indicated that the hemopexin domain is responsible for this interaction. Binding competition between MMP-13 and ADAMTS-4, -5 or TIMP-3, which also bind to cluster II, further shown that the MMP-13 binding site within cluster II is different from those of ADAMTS-4, -5 or TIMP-3. MMP-13 is therefore co-endocytosed with ADAMTS-5 and TIMP-3 by human chondrocytes. These findings indicate that MMP-13 may play a role on physiological turnover of cartilage extracellular matrix and that LRP1 is a key modulator of extracellular levels of MMP-13 and its internalization is independent of the levels of ADAMTS-4, -5 and TIMP-3.

## Highlights

• ProMMP-13 is constitutively produced and endocytosed by chondrocytes.
• LRP1 is a key modulator of extracellular levels of proMMP-13 and MMP-13.
• ProMMP-13 and MMP-13 directly bind to LRP1 *via* the hemopexin domain.
• Unique sites on LRP1 for MMP-13 binding have been mapped.
• Co-endocytosis of proMMP-13 with ADAMTS-4, -5 and TIMP-3.

## 1 Introduction

The matrix metalloproteinases (MMPs) are a family of zinc-dependent proteolytic enzymes that have the capacity to degrade many protein components of the extracellular matrix (ECM). Their activities are considered to have important roles in ECM turnover during embryogenesis, morphogenesis, normal tissue remodelling and repair, but uncontrolled activities contribute to pathogenesis of diverse diseases associated with the tissue destruction, such as arthritis, cardiovascular disease, cancer, chronic ulcers and fibrosis [1], [2], [3], [4], [5], [6], [7].

MMP-13 (collagenase 3) belongs to the collagenase subgroup of the MMP family because of its structural similarity to MMP-1 (collagenase 1) and its ability to cleave interstitial fibrillar collagens. It was originally cloned from human breast cancer tissue [8]. It is produced and secreted from many cell types as a 60-kDa precursor form (proMMP-13), which can be activated to a 48-kDa form *via* a 50-kDa intermediate by plasmin, MMP-2, MMP-3 and MMP-14 [9], [10]. In addition to fibrillar collagen types I, II and III, MMP-13 cleaves other ECM molecules such as N-terminal non-helical telopeptides of type I collagen [11], gelatins [9], type IV, IX, X, and XIV collagens, large tenascin C, fibronectin [12], aggrecan [13], perlecan [14], fibrillin-1 [15], and osteonectin [16]. The enzyme plays a role in ECM remodelling during foetal bone development [17], post-natal bone remodelling [18], and gingival and foetal skin wound repair [19], [20]. Mice lacking the MMP-13 gene exhibit delayed formation of long bones, further providing evidence for the importance of the enzyme in skeletal development [21], [22]. The enzyme is also considered as a major collagenase in the development of osteoarthritis (OA) because of its elevated expression in human OA cartilage and its effective ability to degrade collagen II fibrils [9], [23], [24]. Further support for this is a study with MMP-13-null mice, whose cartilage was protected from degradation in the surgically induced OA model [25]. The wide range of MMP-13 proteolytic capacity suggests that it is a powerful, potentially destructive proteinase, and therefore its activity needs to be strictly controlled under normal physiological conditions.

The activity of MMP-13 can be regulated at multiple levels, which include transcriptional regulation [26], epigenetic modification [27], post-transcriptional regulation by microRNAs [28], [29], [30], activation of pro-enzymes [9], [10] and inhibition by endogenous inhibitors [9], [10]. In addition, Partridge and co-workers have shown that rat MMP-13 is endocytosed and degraded by osteoblastic cells [31] and this requires a two-step processes, involving a first binding to a 170 kDa cell surface receptor with a high-affinity and it is subsequently endocytosed through low-density lipoprotein (LDL) receptor-related protein 1 (LRP1) [32], [33]. However, Bailey et al. [34] reported that the human orthologue of the 170-kDa receptor, Endo180, did not bind to MMP-13 and therefore molecular mechanism behind MMP-13 endocytosis remains to be elucidated.

LRP1 is a type I transmembrane cell surface receptor consisting of a 515-kDa α-chain containing the extracellular ligand-binding domains and a non-covalently associated 85-kDa β-chain containing a transmembrane domain and a cytoplasmic tail. Both chains are derived from a single chain of ~ 600 kDa that is processed by furin during the secretory process. LRP1 is widely expressed [35], [36] and internalizes more than 40 ligands from the extracellular environment, including lipoproteins, ECM proteins, growth factors, cell surface receptors, proteinases, proteinase inhibitors and proteinase-proteinase inhibitor complexes [37], [38]. The ablation of the LRP1 gene in mice is embryonically lethal [39], but tissue specific deletion of the LRP1 gene has demonstrated that it protects the vasculature homeostasis and controls β-amyloid precursor protein trafficking, lipid metabolism in adipocytes, and macrophage biology [37]. In cartilage, LRP1 can endocytose the two major aggrecanases ADAMTS-4 [40] and ADAMTS-5 [41] and tissue inhibitor of metalloproteinases (TIMP)-3, an endogenous inhibitor of collagenases and aggrecanases [42], [43]. LRP1 interacts with frizzled-1 and down-regulates the canonical Wnt-β-catenin signalling pathway [44]. It also represses the hypertrophy of chondrocytes during endochondral ossification by removing connective tissue growth factor (CCN-2) [45], [46]. LRP1 is, therefore, an important regulator of skeletal development and maintenance of cartilage homeostasis. Given that MMP-13 plays a key role on ECM remodelling in skeletal development, its endocytosis must be an important for the regulation of proteinase activity and cartilage homeostasis.

In this study, we investigated the molecular mechanism of MMP-13 endocytosis by human normal chondrocytes. We found that human chondrocytes constitutively express and secrete MMP-13, but its extracellular level is downregulated by LRP1-mediated endocytosis. We also show here that MMP-13 directly binds to LRP1 *via* its hemopexin (Hpx) domain (Hpx_MMP-13_) and its binding clusters in LRP1 have been determined. Competition studies between MMP-13 and ADAMTS-4, -5 and TIMP-3 further revealed the selectivity of its interaction with LRP1 and the co-endocytosis of MMP-13 with ADAMTS-4, -5 or TIMP-3.

## 2 Results

### 2.1 MMP-13 is constitutively expressed, secreted and endocytosed by human normal chondrocytes

**Fig. 1:**
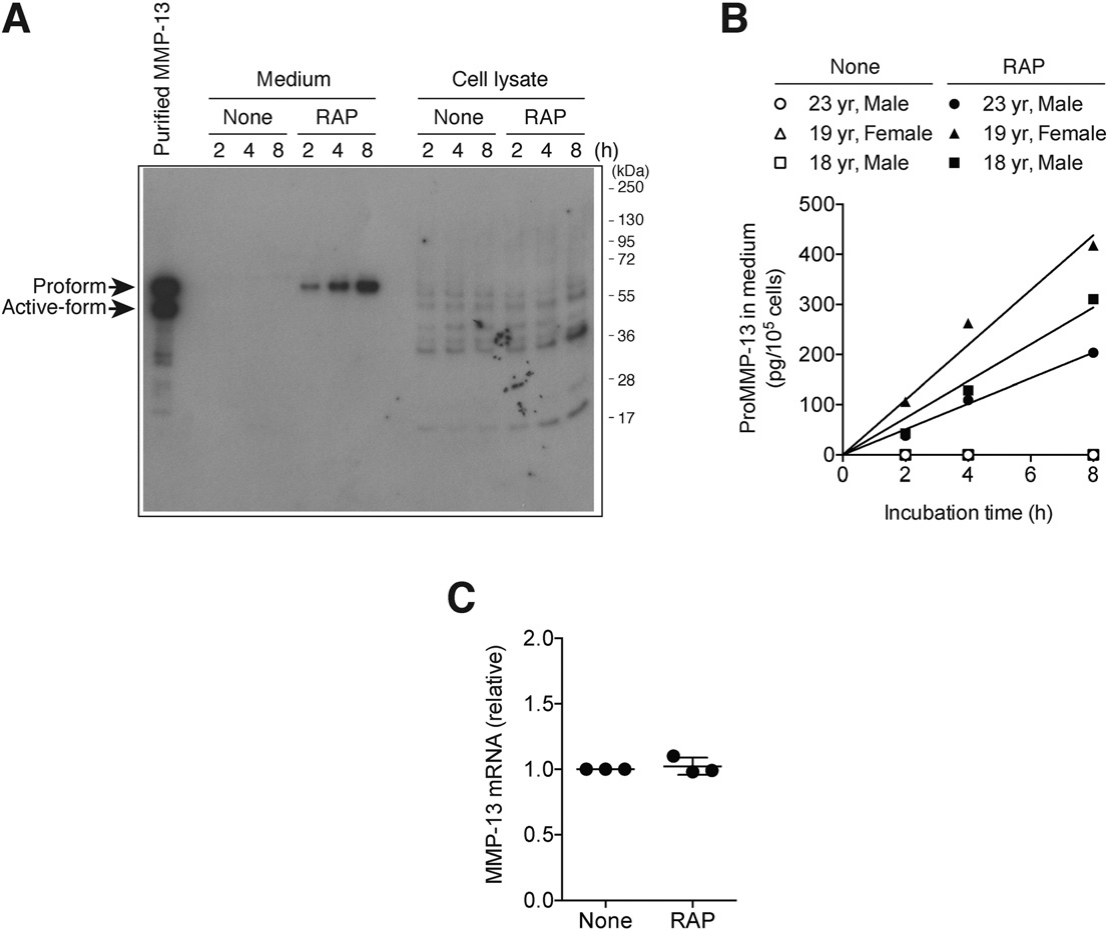
MMP-13 is constitutively expressed, secreted and endocytosed by human normal chondrocytes. Human normal chondrocytes (n = 3) were incubated in the absence or presence of 500 nM RAP for 0–8 h. A, Representative Western blot image for endogenous MMP-13 in the medium and cell lysate detected using an MMP-13 specific antibody (H-230). B, Densitometric analysis of immunoreactive proMMP-13 bands detected in A was carried out. The concentrations of proMMP-13 in the medium were determined in comparison with purified proMMP-13 as described under “Experimental procedures”. C, Results of TaqMan real-time PCR showing relative levels of mRNA for MMP-13 in human normal chondrocytes incubated in the absence or presence of 500 nM RAP for 2 h. *Points* represent the means ± S.D (n = 3).

We first investigated the production of endogenous MMP-13 in human chondrocytes isolated from healthy adults. Endogenous MMP-13 was not detectable by Western blotting in the conditioned medium or the cell lysate of human chondrocytes even after 8-h incubation in the absence of receptor-associated protein (RAP), a ligand-binding antagonist for the LDL receptor family (Fig. 1A). On the other hand, endogenous proMMP-13 was detected in the medium but not in the cell lysate in the presence of RAP and the amount was increased over 8 h (Fig. 1A). Semi-quantitative analysis of immune-reactive bands showed a linear increase of proMMP-13 in media in the presence of RAP over 8 h and that approx. 203–418 pg of proMMP-13 (per 10^5^ cells) accumulated in 8 h (Fig. 1B). We estimated approx. 2.0–4.2 × 10^4^ proMMP-13 molecules were secreted by a single chondrocyte in 8 h, but they were rapidly endocytosed through the LDL receptor family-mediated endocytosis. The quantitative mRNA analysis of human chondrocytes after 2-h incubation in the presence or absence of RAP showed similar levels of MMP-13 mRNA (Fig. 1C).

### 2.2 LRP1 is the primary endocytic receptor for MMP-13 in human normal chondrocytes

**Fig. 2:**
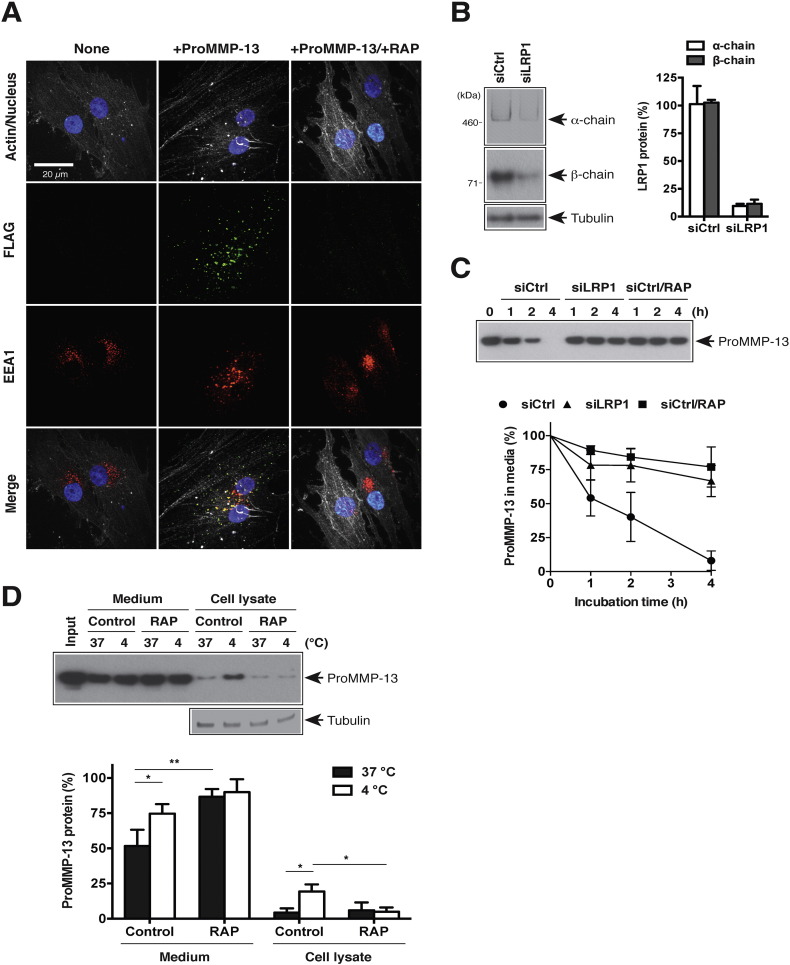
LRP1 is the primary endocytic receptor for MMP-13 in human normal chondrocytes. A, Confocal microscopy analysis of proMMP-13 endocytosis by human normal chondrocytes. Cells were incubated with 20 nM proMMP-13 (FLAG tag at N-terminus) in the presence or absence of 500 nM RAP for 1 h. Endocytosed proMMP-13, EEA1, cytoskeleton, and nucleus were visualized as described under “Experimental procedures”. B and C, Human chondrocytes (n = 3) transfected with non-targeting siRNA (siCtrl) or LRP1 targeting siRNA (siLRP1) were cultured for 2 days in DMEM containing 10% FCS. B, *left panel*, Representative Western blotting for LRP1 α-chain (515 kDa) and β-chain (85 kDa) in cell lysate using anti-LRP1 α-chain (8G1) and β-chain (EPR3724) antibodies, respectively. *Right panel*, Densitometric analysis of immunoreactive LRP1 bands detected was then carried out and the amount of LRP1 was expressed as a % of the amount of LRP1 in untransfected cells (None). C, Human chondrocytes (n = 3) were further incubated with 10 nM proMMP-13 in the absence or presence of 500 nM RAP for 0–4 h and proMMP-13 in the medium was detected by Western blotting using an MMP-13 specific antibody (H-230). *Upper panel*, Representative Western blotting. *Lower panel*, Densitometric analysis of immunoreactive proMMP-13 bands detected in the medium was carried out and the amount of proMMP-13 was expressed as a % of the amount of proMMP-13 at 0 h. D, Human chondrocytes (n = 3) were incubated with 10 nM proMMP-13 in the absence or presence of 500 nM RAP for 1 h at 37 °C or 4 °C, and proMMP-13 in the medium and the cell lysate was detected as in C. *Upper panel*, Representative Western blotting. *Lower panel*, Densitometric analysis of immunoreactive proMMP-13 bands was carried out as in C. *Bars* and *points* represent the means ± S.D. *, *p* < 0.05, **, *p* < 0.01; unpaired t test.

To investigate underlying molecular mechanism of endocytic pathway of MMP-13 in human chondrocytes, we first examined endocytosis of exogenously added proMMP-13 (FLAG tag at N-terminus) by immunofluorescent confocal microscopy. The punctate staining of proMMP-13 was observed within cells, colocalizing with early endosome antigen 1 (EEA1), a marker for early endosomes (Fig. 2A). The intracellular fluorescent signal for proMMP-13 was abolished in the presence of RAP. To investigate whether LRP1 is the primary receptor for the endocytosis of proMMP-13, siRNA-mediated gene silencing of LRP1 was carried out. Silencing LRP1 in human normal chondrocytes by LRP1-targeting siRNA reduced levels of the 515-kDa extracellular α-chain and the 85-kDa β-chain containing the transmembrane domain by 90 and 88%, respectively (Fig. 2B). Cultured chondrocytes endocytosed proMMP-13 rapidly with half-life of approx. 70 min, whereas cellular uptake of proMMP-13 was almost completely inhibited in LRP1-depleted cells (Fig. 2C). We further investigated the role of LRP1 in the surface binding of proMMP-13 by monitoring exogenously added proMMP-13 in culture media and cell lysate at low temperature (4 °C) where endocytosis is blocked. After 1-h incubation with human chondrocytes at 4 °C, ~ 25% of proMMP-13 was detected in the cell lysate whereas only a small portion of proMMP-13 was detected in the cell lysate after 1-h incubation with the cells at 37 °C (Fig. 2D). The increased distribution of proMMP-13 in the cell lysate when cells were incubated at 4 °C was abolished in the presence of RAP.

### 2.3 MMP-13 directly binds to LRP1 *via* its Hpx domain

**Fig. 3:**
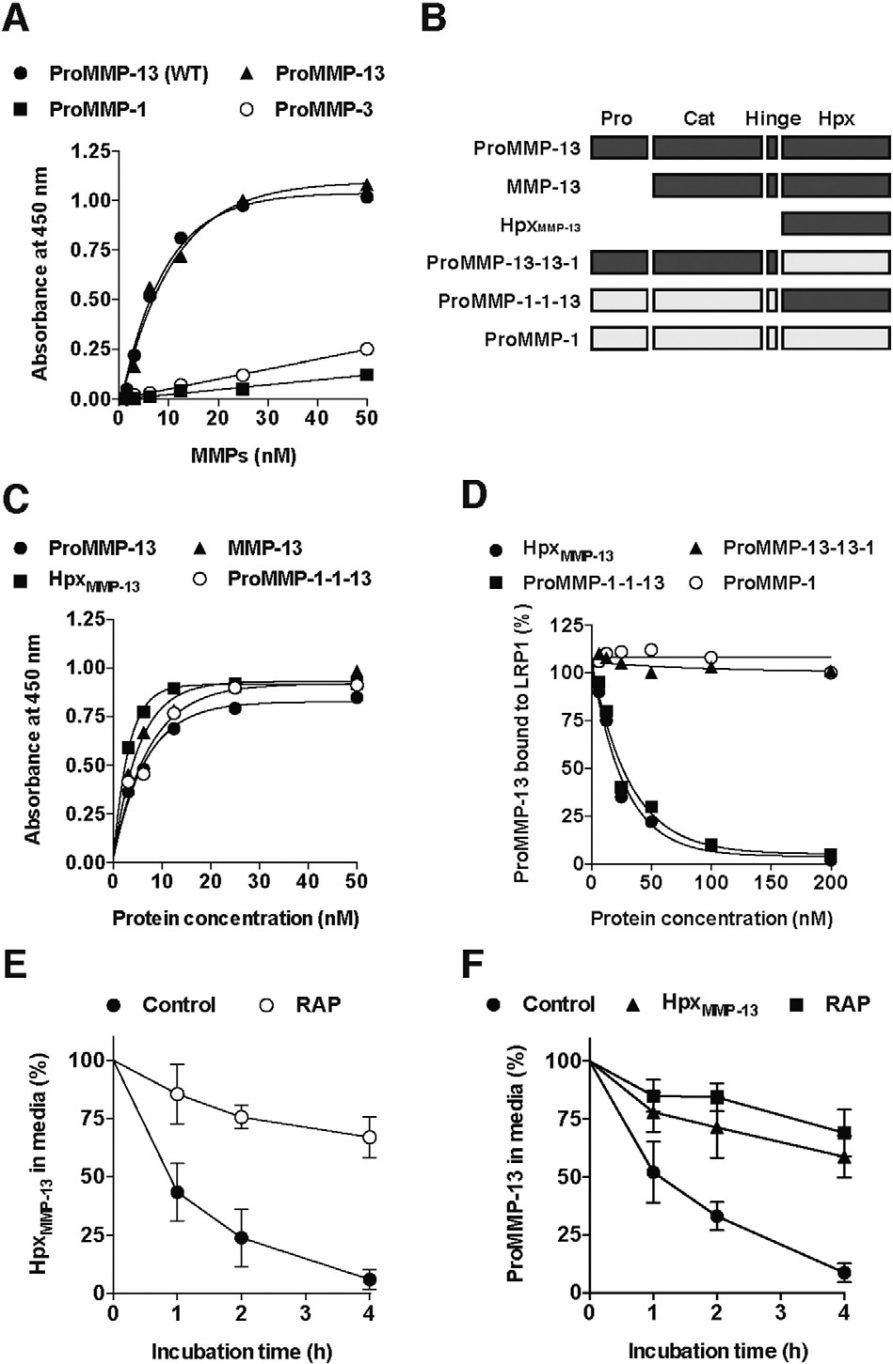
MMP-13 directly binds to LRP1 *via* its Hpx domain. A, Full-length LRP1 was coated onto microtiter plates and binding of proMMP-1(E200A), proMMP-3(E202A), proMMP-13(WT) and proMMP-13(E223A) (each 0–50 nM) was measured using anti-MMP-1 (SB12e), MMP-3 (4B7.3) and MMP-13 (181-14G11) antibodies, respectively, and a horseradish peroxidase-conjugate secondary antibody as described under “Experimental procedures”. B, Schematic representation of MMP-13, its domain deletion mutant and MMP-13/MMP-1 chimeras made by combination of a catalytic domain, a hinge and a Hpx domain from MMP-13 and MMP-1. The MMP-13 sequence is shown as dark grey and the MMP-1 sequence as light grey. *Pro*, pro-domain; *Cat*, catalytic domain; *Hinge*, hinge region; *Hpx*, Hpx domain. C, Full-length LRP1 was coated onto microtiter plates and binding of proMMP-13(E223A), MMP-13(E223A), the Hpx domain of MMP-13 (Hpx_MMP-13_) and proMMP-1-1-13(E200A) (each 0–50 nM) was measured as in A. D, Full-length LRP1 was coated onto microtiter plates and binding of 6 nM proMMP-13(E223A) in the presence of Hpx_MMP-13_, proMMP-13-13-1(E223A), proMMP-1-1-13(E200A) or proMMP-1(E200A) (each 0–200 nM) was measured using an anti-FLAG M2 antibody. The amount of proMMP-13 bound to LRP1 was expressed as a % of the amount of proMMP-13 bound to LRP1 in the absence of the competitor. E, Human chondrocytes (n = 3) were incubated with 10 nM Hpx_MMP-13_ in the absence or presence of 500 nM RAP for 0–4 h, and Hpx_MMP-13_ remaining in the medium was measured as in Fig. 3C. F, Human chondrocytes (n = 3) were incubated with 10 nM proMMP-13(E223A) in the absence or presence of 500 nM Hpx_MMP-13_ or 500 nM RAP for 0–4 h, and proMMP-13 remaining in the medium was measured as in Fig. 2C. *Points* represent the means ± S.D.

We then tested whether proMMP-13 directly binds to LRP1 by a solid-phase binding assay. ProMMP-13 expressed in HEK293 cells and proMMP-13(E223A) expressed in *Escherichia coli* bound to immobilized LRP1 with very similar apparent binding constant (*K*_*D*,*app*_) of 5.9 nM and 6.0 nM, respectively (Fig.3A), whereas proMMP-1(E200A), and proMMP-3(E202A), both of which were expressed in *E. coli*, showed much weaker binding affinities to LRP1. To determine which domain(s) of MMP-13 is responsible for the binding to LRP1, activated forms of MMP-13(E223A) and Hpx_MMP-13_ were analysed. Both MMP-13 and Hpx_MMP-13_ bound to immobilized LRP1 with *K*_*D*,*app*_ values of 3.8 nM and 2.7 nM, respectively (Fig. 3C and Table 1), suggesting that Hpx_MMP-13_ is essential for the interaction with LRP1. To further investigate the role of the Hpx domain in the interaction with LRP1, we constructed MMP chimeras consisting of the catalytic and hinge domains of MMP-13 and the Hpx domain of MMP-1 (MMP-13-13-1(E223A)), or the catalytic and hinge domains of MMP-1 and the Hpx domain of MMP-13 (MMP-1-1-13(E200A)) (Fig. 3B). MMP-1-1-13 bound to immobilized LRP1 with similar affinity to that of MMP-13 (*K*_*D*,*app*_ = 5.1 nM) (Fig. 3C). Furthermore, competition studies on the MMP-13 binding to LRP1 showed that the addition of neither MMP-1 nor MMP-13-13-1 inhibits the MMP-13 binding to LRP1, whereas Hpx_MMP-13_ and MMP-1-1-13 similarly inhibited the binding in a dose-dependent manner (Fig. 3D), supporting our conclusion described above. We further investigated the role of Hpx_MMP-13_ on MMP-13 endocytosis by human chondrocytes. Cultured chondrocytes endocytosed Hpx_MMP-13_ rapidly with half-life of approx. 50 min (Fig. 3E). Furthermore, cellular uptake of proMMP-13 was competitively inhibited by addition of excess amount of Hpx_MMP-13_ (Fig. 3F).

### 2.4 Effect of heparin on the binding of MMP-13 to LRP1

**Fig. 4:**
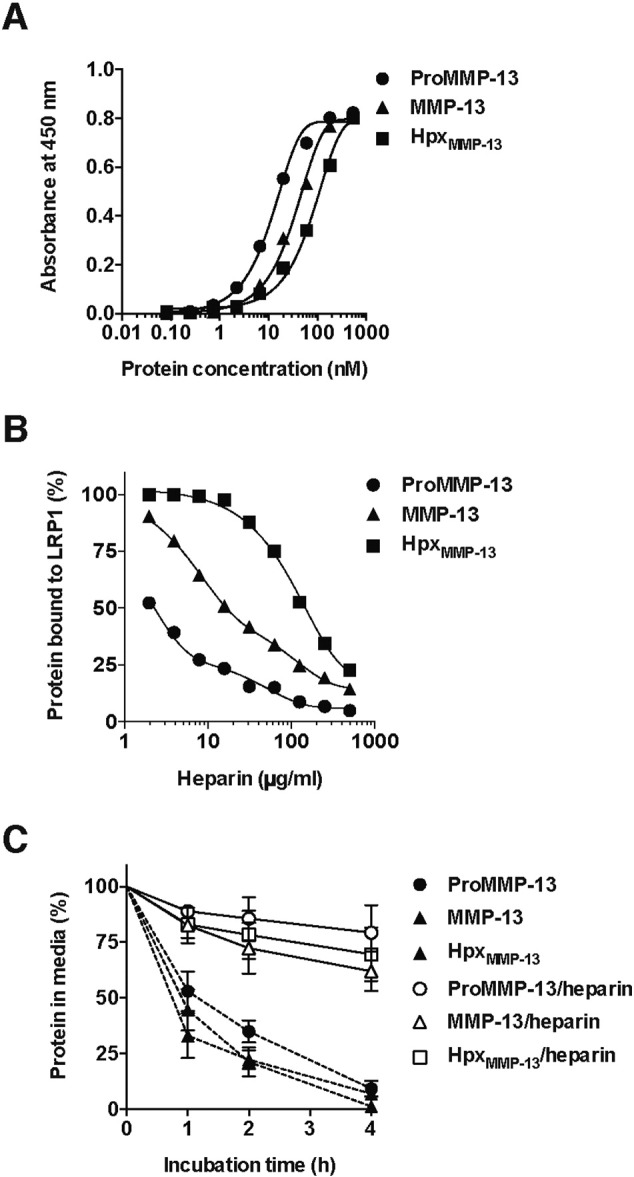
Effect of heparin and type I collagen on the binding of MMP-13 to LRP1. A, Heparin was coated onto heparin binding microtiter plates and the binding of proMMP-13(E223A), MMP-13(E223A) and Hpx_MMP-13_ (each 0–540 nM) was measured using anti-MMP-13 (181-14G11) antibody, and a horseradish peroxidase-conjugate secondary antibody as described under “Experimental procedures”. B, Full-length LRP1 was coated onto microtiter plates and the binding of proMMP-13(E223A), MMP-13(E223A) and Hpx_MMP-13_ (each 3 nM) in the presence of heparin (0–500 μg/ml) was measured as in Fig. 3A. C, Human chondrocytes (n = 3) were incubated with proMMP-13(E223A), MMP-13(E223A) and Hpx_MMP-13_ (each 10 nM) in the absence or presence of 500 μg/ml heparin for 0–4 h, and each protein remaining in the medium was measured as in Fig. 2C. *Points* represent the means ± S.D.

Heparin binds to ADAMTS-4, -5 [47], [48] and TIMP-3 [49], and inhibits endocytosis of these molecules [40], [41], [49]. It has been reported that MMP-13 also binds to heparin [50]. We found that proMMP-13, MMP-13 and Hpx_MMP-13_ bind to immobilized heparin with *K*_*D*,*app*_ of 12 nM, 34 nM and 79 nM, respectively (Fig. 4A and Table 1). We thus examined the effect of heparin on the binding of MMP-13 to LRP1. The binding of proMMP-13(E223A) and MMP-13(E223A), and Hpx_MMP-13_ was inhibited by heparin in a dose-dependent manner with concentrations of heparin that inhibit 50% binding of 2.1 μg/ml, 18 μg/ml and 145 μg/ml, respectively (Fig. 4B). We further investigated the effect of heparin on MMP-13 endocytosis in human chondrocytes. Cellular uptake of proMMP-13, MMP-13 and Hpx_MMP-13_ was almost completely inhibited in the presence of heparin (Fig. 4C).

### 2.5 Binding of MMP-13 to LRP1 ligand binding clusters

**Fig. 5:**
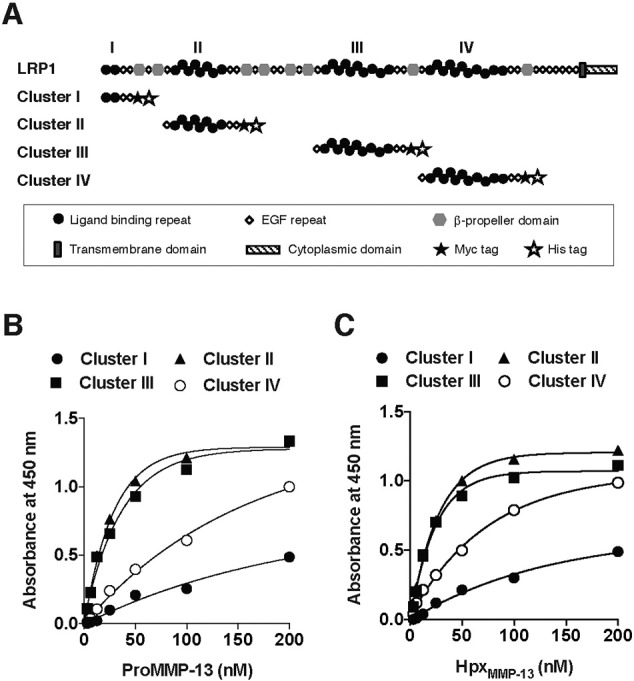
Binding of MMP-13 to LRP1 ligand binding clusters. A, Modular domain organization of LRP1 and its soluble receptor fragments used in this study. The four clusters of ligand-binding clusters are numbered I–IV. The symbols for the various domains are indicated in the inset. B and C, Purified LRP1 fragments (clusters I to IV) were coated onto microtiter plates, and the binding of proMMP-13(E223A) (B) or Hpx_MMP-13_ (C) (each 0–200 nM) was measured as in Fig. 4A.

The ligand binding regions in LRP1 occur in four clusters (clusters I–IV) containing between 2 and 11 individual ligand-binding cysteine-rich repeats (Fig. 5A). To identify the region(s) of LRP1 that binds to MMP-13, each recombinant cluster was coated on multi-well plates and subsequent binding of MMP-13 was quantified. ProMMP-13(E223A) bound to immobilized clusters II and III with high affinity, with *K*_*D*,*app*_ values of 17 nM and 24 nM, respectively (Fig. 5B and Table 2). Likewise, Hpx_MMP-13_ bound to immobilized clusters II and III with high affinity, with *K*_*D*,*app*_ values of 15 nM and 13 nM, respectively (Fig. 5C and Table 2).

### 2.6 MMP-13 does not inhibit the interaction between LRP1 and ADAMTS-4 or -5, or TIMP-3

**Fig. 6:**
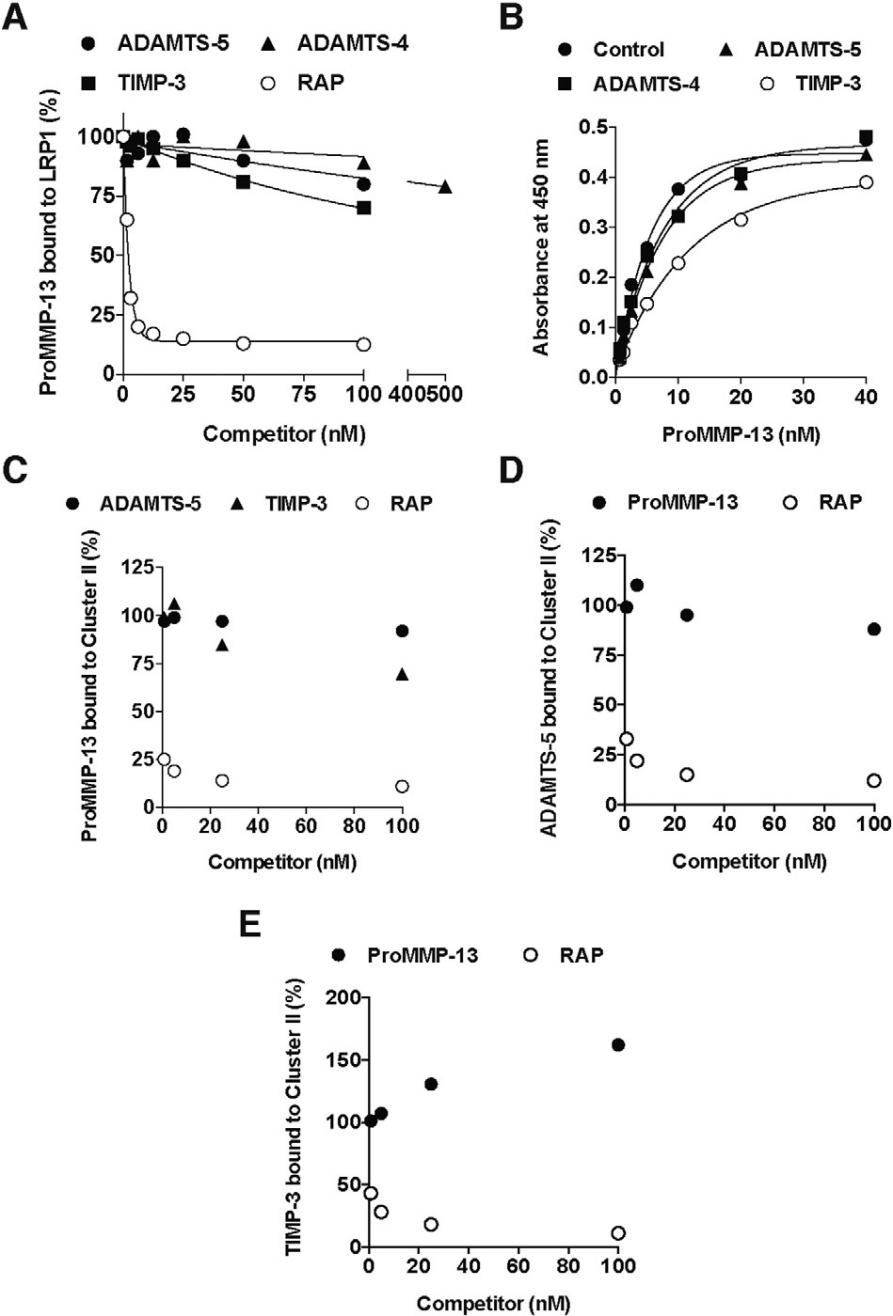
MMP-13 does not inhibit interaction between LRP1 and ADAMTS-4 or -5, or TIMP-3. A, Full-length LRP1 was coated onto microtiter plates and binding of 6 nM proMMP-13(E223A) in the presence of ADAMTS-5, TIMP-3 or RAP (each 0–100 nM), or ADAMTS-4 (0–500 nM) plus 50 μM CT1746 was measured as in Fig. 3A. The metalloproteinase inhibitor CT1746 was added to block autodegradation of ADAMTSs. The amount of proMMP-13 bound to LRP1 was expressed as a % of the amount of proMMP-13 bound to LRP1 in the absence of the competitor. B, Full-length LRP1 was coated onto microtiter plates and the binding of proMMP-13(E223A) (0–40 nM) in the absence or presence of 50 nM ADAMTS-5, 250 nM ADAMTS-4 or 50 nM TIMP-3 plus 50 μM CT1746 was measured as in Fig. 3A. C, Purified cluster II was coated onto microtiter plates, and the binding of 20 nM proMMP-13(E223A) in the presence of ADAMTS-5, TIMP-3 or RAP (each 0–100 nM) plus 50 μM CT1746 was measured as in Fig. 3D. D and E, Purified cluster II was coated onto microtiter plates, and the binding of 5 nM ADAMTS-5 (D) or 5 nM TIMP-3 (E) in the presence of proMMP-13(E223A) or RAP (each 0–100 nM) plus 50 μM CT1746 was measured as in Fig. 3D using anti-FLAG M2 antibody, and a horseradish peroxidase-conjugate secondary antibody as described under “Experimental procedures”. The amount of ADAMTS-5 or TIMP-3 bound to the cluster II was expressed as a % of the amount of ADAMTS-5 or TIMP-3 bound to the cluster II in the absence of the competitor.

To investigate the selectivity of MMP-13 binding sites on LRP1, competition studies were carried out on proMMP-13(E223A) binding to LRP1. The addition of ADAMTS-4, -5, or TIMP-3 did not inhibit proMMP-13 binding to LRP1 (Fig. 6A). ProMMP-13 bound to immobilized LRP1 in the presence of 50 nM ADAMTS-5, 250 nM ADAMTS-4 or 50 nM TIMP-3 with *K*_*D*,*app*_ of 5.2 nM, 5.6 nM and 8.2 nM, respectively (Fig. 6B).

We previously found that ADAMTS-5 binds to clusters II and IV with high affinity, with *K*_*D*,*app*_ values of 3.5 and 9 nM, respectively [40]. We also found that TIMP-3 binds to cluster II with high affinity (unpublished observation). To further investigate whether MMP-13 and ADAMTS-5 or TIMP-3 bind to different regions of cluster II, we carried out competition studies on the binding of these proteins. The addition of ADAMTS-5 did not markedly affect the proMMP-13(E223A) binding to cluster II, or *vice versa* (Fig. 6C and D). The addition of TIMP-3 slightly inhibited proMMP-13 binding to cluster II (Fig. 6C), whereas the addition of proMMP-13 slightly increased TIMP-3 binding to cluster II (Fig. 6E).

### 2.7 Co-endocytosis of MMP-13 and ADAMTS-5 or TIMP-3 in human chondrocytes

**Fig. 7:**
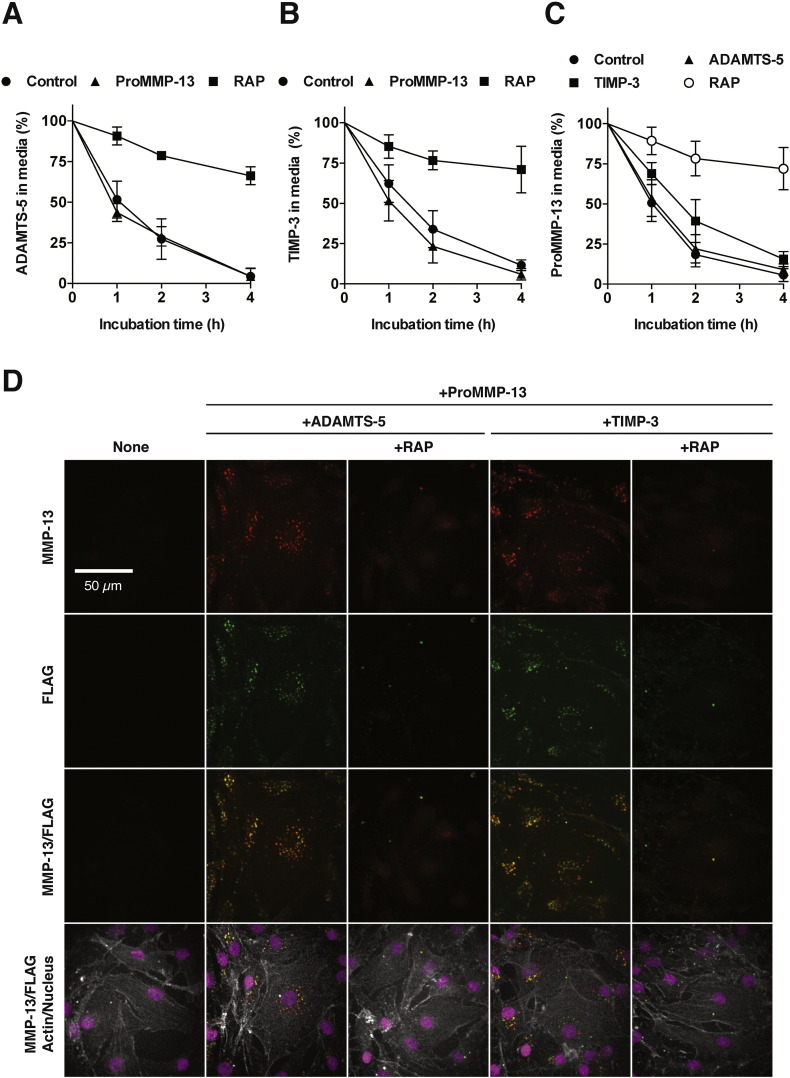
Co-endocytosis of MMP-13 and ADAMTS-5 or TIMP-3 in human chondrocytes. A and B, Human chondrocytes (n = 3) were incubated with 10 nM ADAMTS-5 (FLAG tag at C-terminus) (A) or 10 nM TIMP-3 (FLAG tag at C-terminus) (B) plus 10 μM CT1746 in the absence or presence of 100 nM proMMP-13(E223A) or 500 nM RAP for 0–4 h, and ADAMTS-5 (A) or TIMP-3 (B) remaining in the medium was measured as in Fig. 2C using an anti-FLAG M2 antibody. C, Human chondrocytes (n = 3) were incubated with 10 nM proMMP-13(E223A) plus 10 μM CT1746 in the absence or presence of 100 nM ADAMTS-5, 100 nM TIMP-3 or 500 nM RAP for 0–4 h, and proMMP-13 remaining in the medium was measured as in Fig. 2C. *Points* represent the means ± S.D. D, Confocal microscopy analysis of proMMP-13, ADAMTS-5 and TIMP-3 endocytosis by human normal chondrocytes. Cells were incubated with 10 nM proMMP-13(E223A) and 10 nM ADAMTS-5 or 10 nM TIMP-3 plus 10 μM CT1746 in the absence or presence of 500 nM RAP for 1 h. Endocytosed proMMP-13, ADAMTS-5 and TIMP-3 were visualized using with anti-MMP-13 rabbit polyclonal antibody (H-230) and anti-FLAG M2 mouse monoclonal antibody as described under “Experimental procedures”.

To examine whether MMP-13 competitively inhibits the endocytosis of other molecules such as ADAMTS-5 or TIMP-3, 10 nM ADAMTS-5 or 10 nM TIMP-3 was incubated with human chondrocytes in the presence of 10-fold molar excess of proMMP-13(E223A), and the level of these proteins in the medium was monitored by Western blot analysis. The addition of 100 nM proMMP-13 did not affect the rate of ADAMTS-5 endocytosis (Fig. 7A), whereas the addition of 100 nM proMMP-13 slightly increased the rate of TIMP-3 endocytosis (Fig. 7B). To further test whether MMP-13 endocytosis is competed by ADAMTS-5 or TIMP-3, reverse competition studies were carried out. The addition of 100 nM ADAMTS-5 did not affect the rate of proMMP-13 endocytosis, whereas the addition of 100 nM TIMP-3 slightly reduced the rate of endocytosis (Fig. 7C). We then examined endocytosis of exogenously added proMMP-13(E223A) and ADAMTS-5 (FLAG tag at C-terminus) or TIMP-3 (FLAG tag at C-terminus) by immunofluorescent confocal microscopy. Punctate staining of proMMP-13, ADAMTS-5 and TIMP-3 was observed within cells and most proMMP-13 signals colocalized with the signals of ADAMTS-5 or TIMP-3 (Fig. 7D).

## 3 Discussion

In this study, we have demonstrated that LRP1 is the major endocytic receptor of MMP-13 in human chondrocytes and that it directly binds to MMP-13, mediating its internalization for subsequent lysosomal degradation. This was supported by experiments in which the addition of RAP or gene silencing of LRP1 markedly inhibited the cellular uptake of proMMP-13 from culture media, and by an increased accumulation of proMMP-13 in the cell lysate at lower temperature (4 °C), in a RAP-sensitive manner.

We then identified that the LRP1 binding site of MMP-13 is located solely in the Hpx_MMP-13_ domain. Little contribution to LRP1 binding was made by either the pro-domain or the catalytic domain, since the binding constants of Hpx_MMP-13_ were lower than those of proMMP-13 and the activated form of MMP-13. This finding was unexpected, because MMP-13 has a large positively charged area extending over both the catalytic and in Hpx domains located on the opposite site of the collagen-binding sites [51]. We predicted that these basic residues are most likely involved in the interaction with LRP1, as has been shown for many LRP1 ligands [37]. These residues are also candidates for the binding to heparin and highly sulfated proteoglycans, which interfere the interaction between LRP1 and its ligands, for example, as has been shown for ADAMTS-4 [40], ADAMTS-5 [41], TIMP-3 [42], [49] and for MMP-13 in this study. However, proMMP-13, MMP-13 and Hpx_MMP-13_ bind to heparin with different affinities: the *K*_*D*,*app*_ value for proMMP-13 is 5-fold lower than that of Hpx_MMP-13_, and the inhibitory effect of heparin on proMMP-13 binding to LRP1 is about 70-fold more potent than that for Hpx_MMP-13_. These differences are probably due to the fact that heparin binds to a more extended area of positively charged residues in the proMMP-13 molecule, but the LRP1 binding site is more restricted and localized in the Hpx domain.

There are four ligand-binding clusters in LRP1 (clusters I–IV), each harbouring multiple ligand-binding repeats. Of the LRP1 ligands whose binding sites in LRP1 have been mapped, most of them interact with clusters II and IV [52]. We previously reported that both ADAMTS-4 and -5 bind to clusters II and IV, and showed that ADAMTS-5 is an effective competitive inhibitor of ADAMTS-4 binding to LRP1 as the former has a higher affinity for LRP1 than the latter [40]. These results suggested that these two proteinases share the same binding site on LRP1. In this study, we found that MMP-13 binds to clusters II and III. To date, only four other LRP1 ligands, RAP [53], apolipoprotein E [54], thrombin-protease nexin 1 complex [55], and blood coagulation factor VIII [56] are known to bind to cluster III. Furthermore, we have shown that proMMP-13 does not interfere with the binding of ADAMTS-4 or -5 to LRP1, even though it has high affinity binding constants in the range of 2.7–6.0 nM. This indicates that MMP-13 and ADAMTS-4/-5 bind different sites within cluster II. TIMP-3 also binds to cluster II. Interestingly, the binding affinity of proMMP-13 for LRP1 was slightly reduced in the presence of TIMP-3, whereas TIMP-3 binding affinity to LRP1 was slightly increased in the presence of proMMP-13. Consistent with these observations, the rate of TIMP-3 endocytosis was slightly increased in the presence of proMMP-13, and the rate of proMMP-13 endocytosis was slightly reduced in the presence of TIMP-3. This suggests that proMMP-13 and TIMP-3 bind to different sites on LRP1 but their binding to LRP1 affects the other binding sites allosterically. The distinct binding sites for MMP-13 and for TIMP-3 or ADAMTS-5 on LRP1 are further supported by the co-internalization of proMMP-13 and ADAMTS-5 or TIMP-3 by chondrocytes. Thus, the rate of proMMP-13 endocytosis cannot be greatly altered even when the concentrations of ADAMTS-4, -5 and TIMP-3 are elevated. Once activated, MMP-13 forms a catalytically inactive complex with TIMP-3 [9]. In this case, the Hpx domain of MMP-13 is likely to be outside of the interface of MMP-13 and TIMP-3, and the complex can be endocytosed through both the MMP-13 and TIMP-3 binding sites.

Efficient clearance of MMP-13 by chondrocytes from the extracellular space would explain the generally accepted view that MMP-13 is not produced in most adult human tissues in the steady state. This study has, however, revealed that human chondrocytes isolated from healthy adults constitutively express and secrete proMMP-13, but that this is rapidly endocytosed and degraded by chondrocytes. It is therefore difficult to detect proMMP-13 in the condition medium unless the endocytic pathway is blocked by RAP. Similarly, it is also difficult to detect protein levels of ADAMTS-5 [57] and TIMP-3 [42] in the medium of cultured chondrocytes, even though their mRNAs are present, until their endocytosis is blocked by RAP. We speculate that these molecules including MMP-13, only function for a finite period of time in the turnover of ECM proteins in normal steady state tissues, in order to avoid over-degradation of the cartilage ECM. This also suggests that impairment of the LRP1-mediated endocytosis can disrupt normal tissue homeostasis.

Overexpression of MMP-13 has been observed under numerous pathologic conditions that are characterized by the destruction of collagenous tissue architecture, *e.g.*, in chronic cutaneous ulcers [58], chronic periodontitis [59], atherosclerosis [60], aortic aneurysms [61] and rheumatoid arthritis [62]. The expression of MMP-13 mRNA and its protein levels in cartilage correlate with the progression of OA in humans [23]. Treatment with inflammatory cytokines stimulates MMP-13 expression in human chondrocytes [63]. We previously reported that LRP1-mediated endocytosis is impaired in OA cartilage due to a loss in LRP1 protein without significant changes in its mRNA [41]. A similar impairment of MMP-13 endocytosis was also reported [33]. We therefore proposed that the ectodomain of LRP1 is shed from the cell surface of OA chondrocytes, thereby reducing the endocytic capacity of the cell. Under inflammatory conditions such as in rheumatoid arthritis and systemic lupus erythematosus, MMP-13 transcription is elevated, and LRP1 shedding is also increased [63], [64]. The shedding of LRP1 is also enhanced in chondrocytes treated with interleukin 1 *via* activation of c-jun N-terminal kinase-2 signalling pathway [65]. Thus, an increased shedding of LRP1 along with increased expression of MMP-13 enhances the degradation collagen fibrils in cartilage. Since LRP1 endocytoses not only matrix-degrading proteinases and their inhibitors, but also numerous biologically active factors such as CCN2, transforming growth factor-β, and complement 3C [37], the shedding of LRP1 results in significant alteration of tissue environments and cellular behaviour. We are currently investigating proteinases responsible for the shedding of LRP1 in cartilage as we think they play a key role in initiating a shift of normal chondrocytes to a pathological phase. Identification of such sheddase(s) may help us to develop a new way to control the progression of OA.

## 4 Experimental procedures

### 4.1 Reagents and antibodies

The sources of materials used were as follows: the anti-FLAG M2 mouse monoclonal antibody and heparin from Sigma (Dorset, UK); the anti-EEA1 rabbit polyclonal antibody, the anti-LRP1 α-chain mouse monoclonal antibody 8G1, the anti-LRP1 β-chain rabbit monoclonal antibody EPR3724, the anti-MMP-1 mouse monoclonal antibody SB12e, and the anti-MMP-3 mouse monoclonal antibody 4B7.3 from Abcam (Cambridge, UK); the anti-MMP-13 Hpx domain mouse monoclonal antibody 181-14G11 from Merck Millipore (Darmstadt, Germany); the anti-MMP-13 rabbit polyclonal antibody H-230 from Santa Cruz (Dallas, TX); a hydroxamate-based MMP inhibitor CT-1746 (N1-[2-(S)-(3,3-dimethylbutanamidyl)]-N4-hydroxy-2-(R)-[3-(4-chlorophenyl)-propyl]-succinamide) from UCB Celltech (Slough, UK); a hydroxamate-based MMP inhibitor GM6001 (N-[(2R)-2-(hydroxamidocarbonylmethyl)-4-methylpentanoyl]-L-tryptophan methylamide) from Elastin Product (Owensville, MO); purified human full-length LRP1 from BioMac (Leipzig, Germany). The anti-human ADAMTS-5 catalytic domain rabbit polyclonal antibody was raised in rabbits and characterized [66]. Recombinant human MMP-1(E200A), MMP-3(E202A) [67], MMP-13 with a FLAG tag between the signal and propeptide [68], MMP-13(E223A) [51], ADAMTS-4 lacking C-terminus spacer domain [69], ADAMTS-5 lacking C-terminus thrombospondin domain [66], TIMP-3 [70], RAP [41], and the LRP1 ligand-binding clusters I–IV [40] were prepared as described previously. All other reagents used were of the highest analytical grade available.

### 4.2 Human cartilage tissue preparation and isolation of chondrocytes

Healthy (normal) articular cartilage was obtained from the Stanmore BioBank, Institute of Orthopaedics, Royal National Orthopaedic Hospital, Stanmore from patients following informed consent and approval by the Royal Veterinary College Ethics and Welfare Committee (Institutional approval URN 2010 0004H). Articular cartilage was obtained from the femoral condyles of the knee following amputation due to soft tissue sarcoma and osteosarcoma with no involvement of the cartilage. Tissues were obtained from 5 patients (3 males aged 18, 23 and 57 years; 2 females aged 19 and 68 years). Chondrocytes were isolated as described previously [42]. Both primary and passaged human cells were used in the experiments.

### 4.3 Detection of endogenous MMP-13 in human normal chondrocytes

Cells cultured on 6-well plate were incubated in 2 ml of DMEM with or without 500 nM RAP at 37 °C for 0–8 h. The protein in media was precipitated with TCA and dissolved in 20 μl of 1 × SDS-sample buffer (50 mM Tris-HCl pH 6.8, 5% 2-mercaptoethanol, 2% SDS and 10% glycerol). The cells were washed with DMEM and lysed in 200 μl of 2 × SDS-sample buffer. All samples and various amounts of purified MMP-13 were analysed simultaneously by SDS-PAGE and Western blotting using an MMP-13 specific antibody (H-230). Immunoreactive bands were quantified using ImageJ and the amount of endogenous MMP-13 was determined in comparison with purified MMP-13 within its reasonable linear range.

### 4.4 Quantitative reverse transcriptase-PCR

Quantitative reverse transcriptase-PCR was carried out as described previously [41]. Briefly, cDNA was generated using a reverse-transcription kit (Applied Biosystems, Foster City, CA, USA) and random primers from RNA extracted and prepared using the RNeasy mini kit (Qiagen, Valencia, CA, USA) following the manufacturer's guidelines. cDNA was then used for real time PCR assays using TaqMan technology. The ΔΔ threshold cycle (ΔΔCt) method of relative quantitation was used to calculate relative mRNA levels for each transcript examined. The 60S acidic ribosomal protein P0 (RPLP0) gene was used to normalize the data. Pre-developed primer/probe sets for MMP-13 and RPLP0 were purchased from Applied Biosystems.

### 4.5 Generation of MMP-1/MMP-13 chimera mutants

The constructs of MMP-1/MMP-13 chimeras are shown in Fig. 3B. MMP-1 was cloned into a pET3a expression vector and MMP-1/MMP-13 chimeras were constructed with the overlapping PCR as described previously [71]. MMP-13-13-1(E223A)(MMP-13_1-263_-MMP-1_259-450_) using a sense primer (5′-TCATTGTCGGCATATGCCCCTTCCC-3′) and an antisense primer (5′-ATCAAAGGTTAGCTTACTGTCACATTTGTCTGGCGTTTTTGG-3′) with MMP-13 cDNA in pGEM-T vector as template, and a sense primer (5′-AAACATCCAAAAACGCCAGACAAATGTGACAGTAAGCTAACC-3′) and an antisense primer (5′-GCTTTGTTAGCAGCCGGATCC-3′) with MMP-1 in pET3a as a template. Both PCR fragments were combined in an overlap extension PCR using a sense primer (5′-TCATTGTCGGCATATGCCCCTTCCC-3′) and an antisense primer (5′-GCTTTGTTAGCAGCCGGATCC-3′) 13-UF/pET3a-R, the resulting fragment was cloned into pET3a. MMP-1-1-13(E200A)(MMP-1_1-258_-MMP-13_264-451_) was made using the same method. In short, MMP-1-1-13 construction first the internal NdeI site in the hemopexin domain of MMP-13 was engineered out (CATATG → CCTATG). This MMP-13 construct was then used as a template for PCR with a sense primer (5′-CCAAAAGCGTGTGACCCTTCC-3′) and an antisense primer (5′-GCTTTGTTAGCAGCCGGATCC-3′). A sense primer (5′-TAATACGACTCACTATAGGG-3′) and an antisense primer (5′-GATAAGGAAGGGTCACACGCTTTTGGGG-3′) were used with MMP-1 as a template.

### 4.6 Expression and purification of recombinant proteins

ProMMP-1(E200A), ProMMP-3(E202A), ProMMP-13(E223A), ProMMP-1-1-13(E200A) were expressed, refolded from inclusion bodies and purified as described [71]. ProMMP-13-13-1(E223A) was purified with modifications. After the addition of isopropyl β-D-thiogalactopyranoside, cell culture was continued at room temperature for 5 h. For folding dialysis buffer was 1 mM ZnCl_2_ and 50 mM Tris-acetate (pH 5.5) instead of 50 mM Tris-HCl (pH 7.5) to prevent autolysis. After refolding, samples were applied to an S-column instead of a Green A column equilibrated with 50 mM sodium acetate (pH 5.5), 1 mM ZnCl_2_, 10 mM CaCl_2_, and 0.02% NaN_3_. As final purification step, Sephacryl S-200 column (2.5 × 45 cm) was run using the same buffer. Expression of chimeras using pCEP4/HEK293-EBNA system was essential as described previously [72]. These proteins were detected in the medium, and conditioned medium was harvested twice weekly and the proteins were purified using the method described previously but without Brij-35. The FLAG peptide was removed by gel filtration of Sephacryl S200 equilibrated with Tris-buffered saline (TBS) containing 10 mM CaCl_2_ (TNC) and 0.02% NaN_3_. The hemopexin domain of MMP-13 (Hpx_MMP-13_) was made by incubating proMMP-13 with 1 mM APMA at 37 °C for at least 16 h. The protein was run over a Green A column to remove the full-length MMP-13 and the catalytic domain of MMP-13, and the unbound material containing the Hpx_MMP-13_ was collected.

### 4.7 Immunocytochemical localization of MMP-13, ADAMTS-5 and TIMP-3

Cultured cells on 4-well Lab-Tek chamber slides (Nunc, Roskilde, Denmark) were incubated in DMEM with 20 nM proMMP-13 (with a FLAG tag at N-terminus) in the absence or presence of 500 nM RAP for 1 h at 37 °C. Cells were washed with DMEM, fixed with 3% paraformaldehyde in TNC containing 0.1% Triton X-100 (15 min, room temperature). Each sample was incubated with anti-FLAG M2 mouse monoclonal antibody and anti-EEA1 rabbit polyclonal antibody (3 h, room temperature). Alexa Fluor 488-conjugated anti-mouse IgG and Alexa Fluor 568-conjugated anti-rabbit IgG (Molecular Probes, Eugene, OR) were used to visualize the antigen signals (1 h, room temperature). Actin was stained with Actin-stain 670 phalloidin (Cell Signaling) and nuclei were stained with DAPI. Samples were viewed using a Nikon Eclipse TE2000-U confocal laser scanning microscope. The data were collated using Volocity software (Improvision, Coventry, UK). For co-endocytosis analysis, cultured cells on 4-well Lab-Tek chamber slides were incubated in DMEM containing 10 nM proMMP-13 with or without 10 nM ADAMTS-5 or 10 nM TIMP-3 in the absence or presence of 500 nM RAP for 1 h at 37 °C. Each sample was incubated with anti-MMP-13 rabbit polyclonal antibody (H-230) and anti-FLAG M2 mouse monoclonal antibody (3 h, room temperature). Endocytosed proMMP-13, ADAMTS-5 and TIMP-3, cytoskeleton, and nucleus were visualized as described above.

### 4.8 siRNA knockdown of LRP1 in human articular chondrocytes

siRNA oligonucleotides for LRP1 (On-TargetPlus SMARTpool siRNA) and nontargeting oligonucleotide were purchased from Thermo Scientific Dharmacon (Lafayette, CO). Human articular chondrocytes were plated at a density of 3.5 × 10^4^ cells/well (24-well plate) in DMEM containing 10% FCS and incubated until 50% confluent. INTERFERin (peqlab, Erlangen, Germany) was used to transfect cells with siRNA at a final concentration of 10 nM in Opti-MEM I. At 4 h after transfection, the Opti-MEM was removed and replaced with DMEM containing 10% FCS.

### 4.9 Analysis of endocytic clearance of recombinant proteins

Cells cultured on 24-well plate were incubated in 500 μl of DMEM containing 10 nM of each recombinant protein with or without 500 nM RAP at 37 °C. After incubation for various periods of time, media were collected and the protein was precipitated with TCA and dissolved in 50 μl of 1 × SDS-sample buffer (50 mM Tris-HCl pH 6.8, 5% 2-mercaptoethanol, 2% SDS and 10% glycerol). All samples were analysed by SDS-PAGE and Western blotting using specific antibodies against each recombinant protein. Immunoreactive bands were quantified using ImageJ and the amount of each recombinant protein remaining in the medium at each time point was calculated as a percentage of the amount of each recombinant protein at 0 h.

### 4.10 ELISA for binding of recombinant proteins to LRP1, soluble LRP1 fragments or heparin

Human full-length LRP1 or soluble LRP1 cluster II (5 nM or 25 nM, respectively, in 100 μl of TNC) was coated overnight at 4 °C onto microtiter plates (Corning, NY). Wells were blocked with 3% BSA in TNC (1 h; 37 °C) and washed in TNC containing 0.05% Brij-35 after this and each subsequent step. Wells were then incubated with various concentrations of recombinant proteins in blocking solution for 3 h at room temperature. Bound proteins were detected using each recombinant protein specific antibody (1 h; room temperature) and then with a secondary antibody coupled to horseradish peroxidase (1 h; room temperature). Hydrolysis of tetramethylbenzidine substrate (KPL, Gaithersburg, MA) was measured at 450 nm using a FLUOstar Omega (BMG Labtech). Each value was normalized by subtracting the amount of recombinant protein bound to control the well that was not coated with LRP1 or soluble LRP1 fragments.

For the heparin-binding assay, heparin (10 μg/ml in 100 μl of TNC) was coated overnight at 4 °C onto heparin-binding plates (BD Life Sciences). Wells were blocked with 0.2% (m/v) gelatin in TNC (1 h; 37 °C) and washed in TNC containing 0.05% Brij-35 after this and each subsequent step. Wells were then incubated with various concentrations of recombinant proteins in blocking solution for 3 h at room temperature. Bound proteins were detected using anti-MMP-13 mouse monoclonal antibody 181-14G11 (1 h; room temperature) and then with a secondary antibody coupled to horseradish peroxidase (1 h; room temperature). Hydrolysis of tetramethylbenzidine substrate (KPL, Gaithersburg, MA) was measured as described above.

## Author contribution

KY designed and performed the experiments, analysed the data, and wrote the manuscript. HO, WM, YS and SS performed the experiments and analysed the data. RV designed and generated the expression vectors for MMP-13 and MMP-1/MMP-13 chimera mutants, and purified them. JD prepared human cartilage tissue. LT, DKS, and SH contributed to design the experiments and interpret the results. HN designed the experiments and wrote the manuscript. All authors reviewed the results and approved the final version of the manuscript.

## Statistical analysis

All quantified data represent as the mean ± SD where applicable. Statistical significance was determined by two-tailed unpaired Student's t test, and p < 0.05 was considered significant.

## Declaration of interest

The authors declare no conflict of interest.

## Figures and Tables

**Table 1:** *K*_*D,app*_ values for binding of proMMP-13, MMP-13, Hpx_MMP-13_ to LRP1 and heparin. *K*_*D,app*_ values (nM) were estimated based on the results in Fig. 3, Fig. 4.

	ProMMP-13	MMP-13	Hpx_MMP-13_
LRP1	6.0	3.8	2.7
Heparin	12	34	79

**Table 2:** *K*_*D,app*_ values for binding of proMMP-13, Hpx_MMP-13_, ADAMTS-4 and -5 to LRP1 and its fragments. Extrapolated *K*_*D,app*_ values (nM) were estimated based on the results in Fig. 3, Fig. 5, and those by Yamamoto et al. (40).

	Full-length	Cluster I	Cluster II	Cluster III	Cluster IV
ProMMP-13	6.0	> 200	17	24	> 100
Hpx_MMP-13_	2.7	> 200	15	13	> 50
ADAMTS-4	110	> 1000	240	> 500	330
ADAMTS-5	3.8	> 200	3.5	41	9
